# First stages chronic kidney disease have mild effects on cognitive performance. Results of a 15,105 brazilian adult baseline cohort

**DOI:** 10.1590/1678-4685-JBN-3889

**Published:** 2018-04-26

**Authors:** Valéria Maria de Azeredo Passos, Roberto Marini Ladeira, Cláudia Caciquinho Vieira de Souza, Itamar de Souza Santos, Sandhi Maria Barreto

**Affiliations:** 1 Universidade Federal de Minas Gerais, Belo Horizonte, MG, Brasil.; 2 Secretaria Municipal de Saúde de Belo Horizonte, Belo Horizonte, MG, Brasil.; 3 Hospital Mater Dei, Belo Horizonte, MG, Brasil.; 4 Universidade de São Paulo, Faculdade de Medicina, São Paulo, SP, Brasil.; 5 Universidade Federal de Minas Gerais, Faculdade de Medicina, Belo Horizonte, MG, Brasil.

**Keywords:** kidney diseases, cognition, aging, cardiovascular diseases, doenças renais, cognição, envelhecimento, doenças cardiovasculares

## Abstract

**Introduction:**

The aging of the population may lead to an increased prevalence of dementia and chronic kidney disease (CKD) and their overlap.

**Objective:**

We investigated the association between CKD and cognitive performance among Brazilian adults (35-74 years).

**Methods:**

Baseline data analysis of the Longitudinal Study of Adults (ELSA-Brasil), a multicenter cohort comprising 15,105 civil servants, was performed. Kidney function was defined by the CKD-Epi-estimated GRF and albumin creatinin ratio (ACR). Cognitive performance was measured across tests that included the word memory tests, verbal fluency tests and Trail Making Test B. Multiple logistic and linear regressions were used to investigate the association between CKD and global as well as test-specific lowered cognitive performance.

**Results:**

More than 90% of participants did not present CKD even considering reduced GFR or increased ACR simultaneously. Lowered cognitive performance was detected among 15.8% of the participants and mean values of GFR were slightly higher among those with normal than with lowered cognitive performance (86 ± 15 mL/min/1.73 m^2^ x 85 ± 16 mL/min/1.73 m^2^, *p* < 0.01). Age, education, skin-color, smoking, drinking, hypertension, and diabetes were associated with lowered cognition. After adjustment for these variables, there was no association between CKD and lowered cognitive performance. Negligibly small beta values were observed when analyzing CKD and the scores of all tests.

**Conclusion:**

These results suggest that cognitive performance remains preserved until renal function reaches significant worsening. Preventive measures to maintain renal function may contribute to the preservation of cognitive function.

## Resumo

**Introdução:**

o envelhecimento da população pode levar a uma maior prevalência de demência, doença renal crônica (DRC) e da coexistência dessas doenças.

**Objetivo:**

investigamos a associação entre DRC e desempenho cognitivo em adultos brasileiros (35-74 anos).

**Métodos:**

análise de dados da linha de base do Estudo Longitudinal em Adultos (ELSA-Brasil), uma coorte multicêntrica envolvendo 15.105 funcionários públicos. A função renal foi definida pela TFG estimada CKD-Epi e pela razão albumina/creatinina (RAC). O desempenho cognitivo foi medido em avaliações que incluíram testes de memória de palavras, testes de fluência verbal e Teste de trilhas, versão B (Teste de Trilhas). Regressões logísticas e lineares múltiplas foram usadas para investigar a associação entre DRC e desempenho cognitivo global, bem como desempenho cognitivo reduzido em testes específicos.

**Resultados:**

Mais de 90% dos participantes não apresentaram DRC, mesmo considerando redução da TFG ou RAC aumentada, simultaneamente. O desempenho cognitivo reduzido foi detectado entre 15,8% dos participantes e os valores médios da TFG foram discretamente maiores entre os que apresentam desempenho cognitivo normal (86 ± 15 mL/min 1,73 m^2^ x 85 ± 16 mL/min/1,73 m^2^, *p* < 0,01). A idade, nível educacional, a cor da pele, o tabagismo, o consumo de álcool, a hipertensão e o diabetes estavam associados à cognição reduzida. Após o ajuste para essas variáveis, não houve associação entre DRC e desempenho cognitivo reduzido. Foram observados valores beta insignificantes ao analisar a DRC e as pontuações de todos os testes.

**Conclusão:**

estes resultados sugerem que o desempenho cognitivo permanece preservado até a função renal atingir piora significativa. Medidas preventivas para manter a função renal podem contribuir para a preservação da função cognitiva.

## Introduction

Dementia and chronic kidney disease (CKD) prevalence are expected to increase and their overlap should become more common due to population aging. In middle-income countries such as Brazil, population aging is affected by two factors, the decrease in mortality and increase in life expectancy along with an increasing burden of chronic diseases.^1^ The increasing number of patients with diabetes and cardiovascular disease in addition to the extended survival of individuals with coronary artery disease and stroke are responsible for increasing the prevalence of both cognitive impairment and CKD. Additionally, CKD patients can now achieve substantial longevity due to current improvements in clinical management.^2^

CKD has been associated with worse cognitive performance.^3^^-^ 5 The prevalence of cognitive impairment among patients with end-stage renal disease ranges from 16 to 38% depending on the sample and on the definition of cognitive impairment.^5^ Among menopausal women, a decrease of 10 mL/min/1.73 m^2^ in glomerular filtration rate (GFR) resulted in a 15 to 25% increase in the risk of cognitive impairment.^6^

As cardiovascular risk factors are more frequent in patients with CKD than in the general population, vascular disease probably explains a considerable portion of the association between kidney damage and poor cognitive performance.^7^^-^ 9 However, a detrimental effect of renal impairment on cognitive function may occur independently of cardiovascular disease and their risk factors.^10^^-^ 12

Longitudinal studies have reported poor cognitive function since the initial stages of nephropathy, while worsening of the condition with increasing CKD severity suggests a causal relationship. A meta-analysis of cross-sectional and longitudinal studies comprising 54,779 participants suggested that CKD is a significant and independent risk factor for the development of cognitive decline. The complications of CKD such as water retention, worse blood pressure control, increase in inflammatory cytokines, anemia, and neurotoxicity due directly or indirectly to the progressive accumulation of uremic toxins may be possible mediators of this effect.^13^

In order to establish preventive measures for cognitive and functional performance, it is crucial to understand the role of kidney damage as an independent risk factor for cognitive impairment. As older individuals present common risk factors for both kidney disease and cognitive impairment, many confounders must be considered to disentangle the association between these two conditions among the elderly. By analyzing younger adults, we aimed to clarify the independent association between CKD and cognitive performance.

This study aimed to investigate the association between renal function impairment and cognitive performance using the baseline data from the Brazilian Longitudinal Study of Adult Health (ELSA-Brasil), a cohort comprising predominantly young and middle aged adults.^14^ We hypothesized that worse renal function is associated with lowered cognitive performance independently of social, demographic or clinical factors.

## Methods

This cross-sectional analysis used baseline data of ELSA-Brasil, a cohort designed to identify risk factors and study the natural history of diabetes and cardiovascular disease. ELSA-Brasil comprises 15,105 public civil servants from six Brazilian centers (universities or research institutions), aged 35-74 years at baseline (2008-2010), mostly young adults (78% aged < 60 years), and 54% females.^14^

The study protocol was approved at all six centers by their research ethics committees. After signing an informed consent form all participants were interviewed and examined. The questionnaires included a wide range of social and biological items, as well as a self-reported morbidity section. Participants brought their prescriptions and packages of any medications they had been using in the previous two weeks.^14^

A 12-hour urine sample and a 12-hour fasting blood sample were collected for laboratory tests. The strategies for collection, processing, transportation, and quality control of blood and urine tests in the ELSA-Brasil are described in detail elsewhere.^15^

Cognitive function was determined by standardized tests that assess memory, language, and executive and visual spatial functions. Learning was assessed using the Word Memory Test (WMT), which involves remembering ten unrelated words presented three times, each time for 2 seconds and in a different order. Memory retention was assessed 5 minutes later by free recall. Verbal fluency tests (VTF) consisted in asking participants to say in one minute as many names of animals as possible (semantic test) or words initiated by the F letter (phonemic test). For the trail making B test (Trail B), the participant was instructed to draw lines connecting letters and numbers in alternate order and in an ascending pattern (1, A, 2, B, 3, C, etc.). The participant should draw as quickly as possible, without lifting the pencil point from the page. The final score represents the total time to complete the task, including the time used to make corrections.^16^

We excluded 330 participants who were using medications that potentially interfere with cognition, such as anticonvulsants, antipsychotics, antiparkinsonian, and anticholinesterase drugs, 181 who reported a previous stroke as well as 465 individuals without albumin/creatinine ratio values. The WMT was done by 14,454 (99.0%) participants, as 140 participants, including 21 illiterates, were not able to read. Concerning the VFT, 14,568 (99.8%) and 14,539 (99.6%) participants performed the categorical and phonemic versions, respectively. Trail B was performed by 13,658 (90.4%) participants; 13,160 (90.2%) were able to complete the task in up to five minutes.^17^

Kidney disease was defined based on blood and urine measures of creatinine and albumin. Creatinine was measured in serum specimens by the kinetic Jaffe method (Advia 1200 Siemens, USA), after applying a conversion factor derived from calibration samples traceable to isotope-dilution mass spectrometry. Serum creatinine values were employed to estimate GFR using the C*hronic Kidney Disease Epidemiology Collaboration (*CKD-EPI) equation. Albumin/creatinine ratio (ACR) was calculated from albumin and creatinine concentrations found in the 12-hour urine samples. Urine creatinine was measured by the kinetic Jaffe method and urine albumin by the immunochemical assay (BN II Nephelometer Siemens Dade Behring, USA). Kidney disease was classified using three criteria: 1) an estimated GRF < 60 mL/min/1.73m^2^, 2) an ACR > = 30 mg/g or 3) the combination of these two parameters.^18^

Hypertension was defined as the use of hypertensive medications, systolic blood pressure ≥ 140 mmHg or diastolic blood pressure ≥ 90 mmHg. Diabetes was defined by medical history, the use of antidiabetic drugs, a fasting glucose ≥ 7.0 mmol/L, glycated hemoglobin (HbA1C) levels ≥ 6.5% or a 2h-oral glucose tolerance test ≥ 11.10 mmol/L [14]. Moderate drinking was defined as weekly alcohol consumption above 210 grams and 140 grams for men and women, respectively.^14^ Anemia was defined as hemoglobin less than 13.0 g/dL or 12.0 g/dL for men and women, respectively.^19^

Statistical Analysis was done using STATA™ software, version 12.0.^20^ Continuous variables are described by medians, means and standard deviations. The categorical variables are described by percentages.

We used multiple logistic regressions to determine the association between CKD and lowered cognitive performance. A composite z-score was obtained by adding the z-scores of memory and verbal fluency tests and subtracting the Trail Making B Test z-scores. Given the influence of age and education on the performance of the cognitive tests, the subjects were stratified into three age groups (35-44, 45-64 and 65 + years) and into four levels of education (< 8, 8-10, 11-14 and 14 + years of schooling). For each stratum, the mean and standard deviation (SD) of composite z-scores of cognitive function were calculated and global lowered cognitive performance was defined as one or more SD below average in each age and education stratum.^17^ The same strategy was used to obtain the lowered cognitive performance on each test.

Additionally, we used multiple linear regression analysis to determine the association between kidney dysfunction (according to the three aforementioned definitions) and the crude scores of each cognitive test. The crude score of Trail B was log transformed due to its skewed distribution before performing the linear regression.

Both multivariate logistic and regression analyses were sequentially adjusted by the following potential confounders: 1) socio-demographics: sex, age, skin color, and schooling; 2) behavioral: alcohol use and smoking status; and 3) clinical: hypertension, diabetes, and anemia.

## Results

The participants of this study are similar to the original cohort, after applying the exclusion criteria^21^. Given the nature of the sample, there was a small predominance of women (54.8%), the majority are middle-aged adults (mean = 51.6 + 9.0 years-old), and they have a higher education level than the Brazilian population (over half of the participants had a university degree). Fifty-three point five percent declared themselves white. Current smoking and alcohol drinking were reported by 12.8 and 70.9% of participants. The frequency of hypertension, diabetes and anemia in the sample was 34.2, 18.6 and 5.2%, respectively. Lowered cognitive performance was detected among 15.8% of the participants and mean values of GFR were slightly higher among those with normal compared with lowered cognitive performance (86 ± 15 mL/min/1.73 m^2^ *vs.* 85 ± 16 mL/min/1.73 m^2^, *p* < 0.01). More than 90% of participants did not present CKD even considering reduced GFR or increased ACR simultaneously.

On bivariate analysis, lowered cognitive performance was more prevalent among older participants, smokers and participants with brown and black skin color. The prevalence of lowered cognitive function was also greater among participants with hypertension, diabetes, and CKD, regardless of the adopted definition (Table 1).

**Table 1:** Characteristics of the cohort at baseline (n = 13,658)

	Cognitive performance	o
	Normal	
Age, year (mean, SD)	51.4 (8.9)	52.8 (9.2) **
Sex (female, %)	56.0	45.8 *
Race/Skin color (%)		
White	55.0	45.1
Black	14.4	18.6 **
Brown	27.4	31.5 **
Asian	2.5	3.2 **
Indigenous	0.8	1.6 **
eGFR (CKD-EPI) - mL/min/173m^2^ (%)		
eGFR > 90	41.3	38.3
eGFR 60-89	54.6	55.5
eGFR 45-59	3.6	5.2 **
eGFR 30-44	0.4	0.7*
eGFR < 30	0.1	0.3
Albuminuria (ACR > = 30%)	4.5	5.8**
CKD (GFR < 60 and/orACR > = 30) (%)	7.9	1.
Smoking (%)	12.0	1
Alcohol drinking (%)	71.0	1.
Moderate	64.0	58.3
Excessive	▪	6.9
Anemia (%)	▪	5.2
Hypertension (%)	33.2	39.3 **
Diabetes (%)	17.7	23.2 **
Cognitive test scores (median, IQR)		
Immediate memory	22 (20-25)	17 (15-19)
Recall memory	8 (6-9)	5 (4-6)
Phonemic VFT	13 (11-16)	9 (7-12)
Semantic VFT	19 (16-23)	15 (12-17)
Trail B Test	92 (71-127)	143. (99-228)

eGFR: estimated glomerular filtration rate, CKD: chronic kidney disease, ACR: albumin/creatinine ratio; SD: standard deviation, IQR: interquartile range, VFT: Verbal Fluency Test; Multivariate analysis:

* *p* < 0.05;

** *p* < 0.01.

After adjusting for confounders, there was no association between lowered cognitive performance and CKD. Additionally, kidney dysfunction was associated with a very small increase in the chance of lowered cognitive performance in this population, with negligible modifications after adjusting for social demographic, behavioral, and clinical variables (Table 2). Poor kidney function and lowered cognitive performance were not associated also when comparing each test separately (data not shown).

**Table 2:** Multiple logistic regression analysis of the global cognitive performance according to kidney function. ELSA-Brasil cohort study, 13,658 participants, 2008-2010

	Model 1	Model 2	Model 3
	OR (CI 95%)	OR (CI 95%)	OR (CI 95%)
Glomerular Filtration Rate			
≥ 60 mL/min/1,73m^2^	1.0	1.0	1.0
< 60 mL/min/1,73m^2^	1.23 (1.00-1.52)	1.22 (0.99-1.51)	1.20 (0.97-1.49)
Albumin/creatinine ratio			
< 30 mg/g	1.0	1.0	1.0
≥ 30 mg/g	1.10 (0.89-1.36)	1.09 (0.88-1.35)	1.05 (0.85-1.31)
GFR+ACR^a^			
No	1.0	1.0	1.0
Yes	1.16 (0.99-1.37)	1.15 (0.98-1.36)	1.13 (0.95-1.33)

• GFR: glomerular filtration rate + ACR: albumin/creatinine ratio; Model 1: adjusted by sociodemographic variables (sex, age, race); Model 2: adjusted by sociodemographic and behavioral (smoking and drinking); Model 3: adjusted by sociodemographic, behavioral and clinical (hypertension, diabetes and anemia).

Table 3 shows the association of CKD with the crude scores of each cognitive test after adjustments. Semantic VFT was not associated to any classification of CKD. The scores on immediate memory test decreased when proteinuria was detected. The scores on recall memory test and Phonemic VFT decreased when GFR was lower than 60 mL/min/1.73m^2^ (Table 3). As expected, the combination of decreased GFR and/or increased ACR was associated with a poor performance in the same domains observed for the two isolated criteria (data not show). The coefficients of determination (R^2^) were very similar for the models including all variables except CKD for all cognitive domains (Table 3). When the analysis was done by age strata (35-44, 45-64 and 65 + years), we found no association of cognition and CKD (data not show).

**Table 3:** Multiple linear regression analysis of cognitive tests performance by kidney dysfunction. ELSA-Brasil cohort study, 13658 participants, 2008-2010

CHRONIC KIDNEY DISEASE						
	Lower GFR		High ACR		Lower GFR+High ACR	
	Beta* (95% IC)	R^2^	Beta* (95% IC)	R^2^	Beta*	R^2^
Global cognitive performance	-0.07 (-0.1; -0.02)	0.40	-0.04 (-0.08; 0.002)	0.40	-0.05 (-0.09;-0.02)	0.40
Immediate memory	-0.29 (-0.59; 0.01)	0.18	-0.30 (-0.59;-0.01)	0.18	-0.27 (-049; -0.15)	0.18
Recall memory	-0.22 (-0.38; -0.07)	0.16	-0.06 (-0.21; 0.08)	0.16	-0.15 (-0.27; -0.04)	0.16
Semantic VFT	-0.20 (-0.60; 0.19)	0.19	0.11 (-0.26; 0.50)	0.19	-0.05 (-00.35; 0.24)	0.19
Phonemic VFT	-0.54 (-0.89; -020)	0.13	-0.12 (-0.46; 0.21)	0.13	-0.33 (-0.60; -0.07)	0.13
Trail B test	0.78 (-3.40; 4.97)	0.40	4.16 (0.08; 8.24)	0.40	2.17 (-0.99; 5.33)	0.40

* After adjustment by sex, age, race, education, smoking, drinking, and the presence of hypertension, diabetes or anemia; VTF: Verbal Fluency Tests.

The multivariate analysis by age strata (35-44, 45-64 and 65 + years) showed an association between lower GFR and lowered global cognitive performance among the middle-aged (OR = 1.60, 95% CI 1.22-2.09), but not among elderlies. The combination of lower GFR and increased ACR was associated with lowered global cognitive performance among middle-aged and elderly, but the lower limit of the confidence interval close to 1.0 (table 4). We found similar results when analyzing data by specific cognitive tests (data not shown).

**Table 4:** Multiple logistic regression analysis of the global cognitive performance, according to kidney function, by age group

	35-44 yrs N = 3166	45-64 yrs N = 9003	65-74 yrs N = 1267
	OR (CI 95%)	OR (CI 95%)	OR (CI 95%)
GFR*			
≥ 60 ml/min/1.73m^2^	1.0	1.0	1.0
< 60 ml/min/1.73m^2^	0.74 (0.21-2.61)	1.60 (1.22-2.09)	1.24(0.85-1.79)
ACR*			
< 30 mg/g	1.0	1.0	1.0
≥ 30 mg/g	0.94 (053-1.68)	1.01 (0.77-1.32)	1.54(0.94-2.52)
GFR + ACR*			
No	1.0	1.0	1.0
Yes	0.86 (0.50-1.49)	1.23 (1.00-1.51)	1.49(1.05-2.10)

* After adjustment by sex, race, smoking, alcohol drinking, hypertension, diabetes, and anemia.

## Discussion

Our results suggest that cognitive performance is maintained until renal function reaches significant worsening. We used two different approaches to investigate the association of CKD with cognitive performance. First, considering the high influence of education on the performance of the tests in this Brazilian population, we used standardized z-scores to control the effect of age and education on tests’ scores and defined low cognitive performance as one SD bellow the mean.^17^^,^ 22 This analysis failed to demonstrate the association of CKD with global lowered cognitive performance or lowered cognitive performance on each cognitive test, after adjustment for well-established risk factors for cognitive performance and CKD. The second strategy analyzed the influence of each definition of CKD on crude scores of each cognitive test and we found a modest impact of CKD on cognitive performance.

The relatively young age of ELSA-Brasil participants may explain the lack of association between CKD and lowered cognitive performance. A study comprising 4,095 participants also including young people (aged 35 to 82 years) found no association of GFR with cognitive function, although elevated albuminuria was associated with worse cognitive function only among the young on prospective analysis. Lower GFR was significantly associated with lower cognitive function when the analysis was limited to elderly (65 + years old) with high cardiovascular risk.^23^ Moderate CKD (GFR < 50 mL/min per 1.73 m^2^) was associated to an increased prevalence of cognitive impairment, independent of confounding factors, when analyzing 23,405 individuals that were older (mean age 64.9 ± 9.6 years) and presented higher prevalence of CKD (11%) than our participants.^24^

The low prevalence of moderate to severe CKD among the ELSA-Brasil participants may also contribute to the modest association between CKD and cognitive performance. Severity of CKD seems to influence the existence and the degree of association between renal function and cognitive performance. A study comprising 4,849 healthy young individuals (20-59 years old) showed an association between stage 3 CKD and lower cognitive function independently of confounders.^25^ Another cross-sectional study comprising 825 CKD adults (55+ years old) found that the risk of impairment on global cognition and on the majority of the cognitive domains is higher below the GFR threshold of 30 mL/min/1.73 m^2^, reported as twice as that observed in individuals with GFR between 45 and 60 mL/min/1.73 m^2^.^26^ Additionally, a meta-analysis of seven cross-sectional studies revealed that CKD severity was significantly associated with lowered cognitive performance in a dose-response relationship. A higher odds ratio was observed for severe CKD than for moderate or mild CKD.^13^ Four out of ten longitudinal studies failed to show an increased risk of cognitive decline with GFR < 60 mL/min/1.73 m^2^ and an association was detected only in the presence of moderate to severe CKD in other 2 studies.^13^ Methodological differences regarding sex, age, methods of definition of CKD, and choice of cognitive tests certainly account for the variability on studies results.^13^

Although modestly, GFR was significantly associated with a worst cognitive performance in recall memory and Phonemic VFT, while proteinuria was associated with learning memory. Albuminuria and low GFR seem to be complementary, but not additive risk factors for incident cognitive impairment. 27 In this study, the association of distinct cognitive domains with impaired levels of GFR or proteinuria reinforces this hypothesis.

The main strengths of this study rely on the rigorous methodology, the great number of participants and the wide age range, but the cross-sectional design and the lack of severe CKD cases limited the analysis.

## Conclusion

Our results suggest that cognitive performance is maintained until a significant deterioration of renal function occurs. Cross-sectional studies show conflicting results on association between cognitive impairment and kidney dysfunction, but most of the significant associations only occur with the final stages of renal dysfunction. As CKD results from many health problems that damage the kidney generally by a progressive and chronic progression, preventive and therapeutic strategies could slow the loss of kidney function and consequently protect cognitive function. ELSA-Brasil follow-up will investigate predictors for cognitive dysfunction among adults with initial levels of CKD aiming to obtain effective preventive measures.
